# A Case of Severe Lactation Ketoacidosis in a Nondiabetic Mother on a Ketogenic Diet

**DOI:** 10.1210/jcemcr/luad134

**Published:** 2023-11-07

**Authors:** Marisa Khatijah Borhan, Shireene Ratna Vethakkan, Tharsini Sarvanandan, Sharmila Sunita Paramasivam

**Affiliations:** Department of Medicine, University Malaya Medical Centre, 59100 Kuala Lumpur, Malaysia; Department of Medicine, University Malaya Medical Centre, 59100 Kuala Lumpur, Malaysia; Department of Medicine, University Malaya Medical Centre, 59100 Kuala Lumpur, Malaysia; Department of Medicine, University Malaya Medical Centre, 59100 Kuala Lumpur, Malaysia

**Keywords:** lactation, ketoacidosis, ketogenic diet, breastfeeding

## Abstract

Lactation ketoacidosis is a rare yet severe metabolic emergency that has been reported in breastfeeding mothers. Reduced carbohydrate intake during breastfeeding has been reported as a common trigger for ketoacidosis. We report the case of a 31-year-old mother without diabetes who presented with life-threatening lactation ketoacidosis after following a ketogenic diet while exclusively breastfeeding her newborn baby. She was managed in the intensive care unit with dextrose and insulin infusion to reverse ketoacidosis. With prompt treatment, the patient's ketoacidosis resolved within 24 hours, and she was discharged well 3 days later. We further discuss the underlying increased metabolic demand in lactating women that puts them at risk of ketoacidosis, underlining the importance of early recognition of lactation ketoacidosis and nutritional education for lactating women.

## Introduction

Lactation ketoacidosis (LKA) is an uncommon phenomenon, with fewer than 30 cases reported in the literature to date [1-8]. In lactating women, a mismatch between increased metabolic demand and reduced carbohydrate intake can precipitate ketoacidosis. Almost all reported cases were precipitated by a low-carbohydrate diet during lactation [8]. “Ketogenic diets” have been popularized recently because of their potential for rapid weight loss. Consequently, these diets result in a ketogenic state secondary to very low or absent carbohydrate intake, thus posing a risk to certain high-risk individuals. As new diet regimens and fads continue to emerge without proper diet counseling and monitoring, such complications may become more commonplace. We report a case of severe life-threatening LKA in a nondiabetic, postpartum lactating woman practicing a self-prescribed ketogenic diet.

## Case Presentation

A 31-year-old woman presented to our emergency department at 2 months postpartum with a 1-week history of severe lethargy followed by an acute episode of shortness of breath and epigastric pain. She also reported 1 instance of a fall during that week following a presyncopal episode. She was fully conscious and uninjured after the fall, and therefore did not seek any medical attention. Aside from shortness of breath and epigastric pain, she denied experiencing any other respiratory, cardiac, or gastrointestinal symptoms. She also denied having fever or any history suggestive of deep vein thrombosis or pulmonary embolism.

This was her first child, and she had an uncomplicated pregnancy and childbirth. She did not have a history of diabetes and oral glucose tolerance tests performed twice during pregnancy were normal. She had been well during the postpartum period and was exclusively breastfeeding her child. She started following a ketogenic diet found online at 6 weeks postpartum with the aim of losing the weight she had gained during pregnancy. Her prepregnancy weight and body mass index were 55 kg and 23.2 kg/m², respectively, and she gained 12 kg during pregnancy, reaching a weight of 67 kg before delivery. During the 3 weeks of the ketogenic diet, she had consumed only boiled eggs, salad, and 4 spoonfuls of rice or konjac vermicelli for all her main meals. Her weight was 62 kg before starting the ketogenic diet, and while adhering to it, she had lost 8.8 kg within 3 weeks. She continued to fully breastfeed her child during this period.

## Diagnostic Assessment

On presentation to the emergency department, she was tachypneic with a respiratory rate of 40 breaths/minute and febrile with a temperature of 38 °C. Her blood pressure was 120/80 mm Hg, her heart rate was 92 beats/minute, and her oxygen saturation level was 99% under room air. Clinically, she appeared lethargic and dehydrated. The rest of the physical examination was unremarkable, except for the presence of epigastric tenderness and mildly engorged breasts. Volume status assessment via bedside ultrasound revealed a collapse inferior vena cava during inspiration, which suggested hypovolemia.

Her blood glucose level on arrival was 79 mg/dL (4.4 mmol/L) (normal range [NR]:  72-140 mg/dL;  4.0-7.8 mmol/L). Her blood ketone level was 40.7 mg/dL (7.0 mmol/L; NR: < 3.49 mg/dL; < 0.6 mmol/L). Her arterial blood gas showed severe metabolic acidosis with a pH of 7.096 (NR: 7.35-7.45), a bicarbonate level of 8.0 mEq/L (8.0 mmol/L; NR: 23-29 mEq/L), and base excess of −27.2 mEq/L (−27.2 mmol/L; NR: ± 2.0 mEq/L]. Her renal profile was normal and infection markers were not elevated: white cell count 9300/µL (9.3 × 10^9^/L; NR: 4000-1000/µL; 4.0-10.0 × 10^9^/L) and C-reactive protein .26 mg/dL (2.6 mg/L; NR <.50 mg/dL; < 5.0 mg/L).

## Treatment

A diagnosis of LKA was made, likely precipitated by the ketogenic diet. She was resuscitated with a total of 4 L of normal saline as graded boluses and maintained on 125 mL/hour of dextrose 10% (D10%) drip. Insulin infusion was initiated at 3 U/hour (.05 U/kg/hour) once her blood glucose level increased to more than 144 mg/dL (8 mmol/L). Because of respiratory distress from severe acidosis, she was transiently given noninvasive ventilatory support and monitored in the intensive care unit (ICU).

## Outcome and Follow-up

In the ICU, she was maintained on a D10% drip and insulin infusion. She was allowed to eat and drink as tolerated and was encouraged to express breast milk to relieve her mastitis. Throughout the ICU stay, she remained afebrile. Her infection markers remained low and her blood cultures were sterile; therefore, she did not require antibiotics. She was monitored closely for refeeding syndrome and developed hypophosphatemia with a serum phosphate level of .84 mg/dL (.27 mmol/L; NR: 2.40-5.10 mg/dL; .78-1.65 mmol/L), which was easily corrected with IV phosphate corrections. The serum phosphate level normalized to 3.03 mg/dL (.98 mmol/L) before discharge.

**Figure 1.**
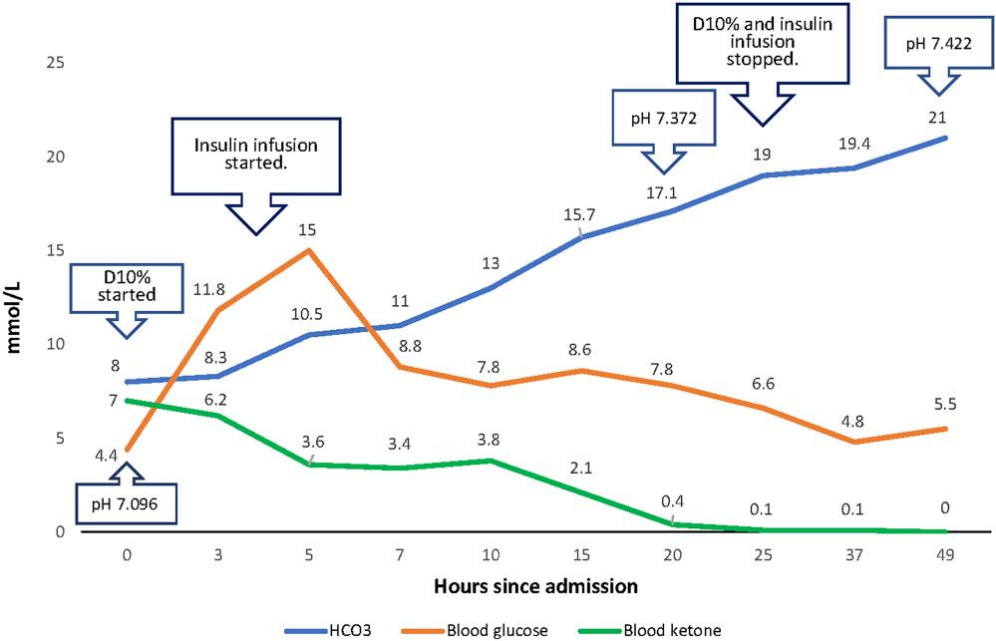
Changes in pH, bicarbonate (HCO3, mmol/L), blood glucose (mmol/L), and blood ketone (mmol/L) levels throughout admission.

She was weaned off noninvasive ventilatory support within 4 hours of ICU admission because her acidosis continued to improve. Within 20 hours of presentation, her blood gases and blood ketones improved to a pH of 7.372, a bicarbonate of 17.1mEq/L (17.1 mmol/L), and a blood ketone level of 2.3 mg/dL (.4 mmol/L) (Fig. 1). As she started eating well, both the D10% drip and insulin infusion were discontinued. Her blood glucose level remained stable, ranging from 86 to 123 mg/dL (4.8-6.8 mmol/L) after the cessation of D10% and insulin infusion.

The patient was transferred to the general medical ward on day 3. Her glycated hemoglobin level at admission was normal at 4.8% (29 mmol/mol). She was advised by a dietitian on healthy nutrition during lactation. The patient was discharged home on day 4 after a full recovery. She was followed-up at the outpatient clinic 2 weeks after discharge, at which time she was well, with normal blood parameters, including blood glucose.

## Discussion

Ketosis is uncommon in lactating women because humans can adapt to higher calorie demands during lactation by increasing food intake and metabolic adjustments. Unfortunately, prolonged starvation and caloric restriction, either intentional or unintentional, may compromise the health of lactating women.

Ketoacidosis in a lactating woman, termed “lactation ketoacidosis” was first described in 1982. To date, fewer than 30 cases have been reported in the literature [1-8]. None of the reported lactating women had underlying diabetes mellitus [1-8]. In a systematic review of 18 cases, 76% of cases were precipitated by recent dietary modifications, including ketogenic diets [8]. A ketogenic diet is described as a very low carbohydrate (< 50 g/day) and high-fat diet that has been proven to achieve significant weight loss in obese patients. While on a ketogenic diet, the body uses fat for energy metabolism, resulting in the accumulation of ketones in blood and urine [2]. In high-risk individuals, the intended nutritional ketosis can lead to life-threatening ketoacidosis. When comparing different carbohydrate intakes (30% intake vs 60% intake) among lactating women, Mohammad et al reported that milk fat, energy output, and energy expenditure were higher during the lower carbohydrate diet, which led to a greater energy deficit [9]. Therefore, reduced carbohydrate intake as practiced in a ketogenic diet, may put lactating women at risk of ketoacidosis, as demonstrated in our case.

Lactating women require an additional 300 to 500 kcal per day to support milk production [2]. One case reported a woman who was breastfeeding 2 babies while attempting to lose weight and suggested that increased caloric demand while breastfeeding was the contributing factor for LKA [7]. Another study by Mohammad et al compared energy expenditure in lactating vs nonlactating women fasting for varying periods. After 14 hours of fasting, the lactating women had a 30% increase in glucose demands, which was compensated for by increased rates of glycogenolysis [10]. Following 42 hours of fasting, lactating women had a higher rate of gluconeogenesis than nonlactating women because of earlier depletion of liver glycogen stores [10]. Interestingly, the milk volume and macronutrient content remained within the normal range throughout the prolonged fasting [10]. Thus, gluconeogenesis is increased in lactating women to cope with the increase in energy expenditure required for milk production during extended fasting.

Although our patient did not experience hypoglycemia, most of the reported patients with LKA presented with hypoglycemia (median plasma glucose level, 3.8 mmol/L) [8]. During fasting, the body protects against hypoglycemia by lowering serum insulin levels and releasing counterregulatory hormones such as glucagon, cortisol, epinephrine, and growth hormones [2]. This leads to reduced peripheral tissue glucose utilization and glycogenolysis and gluconeogenesis activation. However, the rate of mammary hexoneogenesis remains the same throughout the fasting period because the body prioritizes nutrient delivery to the mammary gland to maintain milk production [10]. The resulting low glucose levels in breastfeeding women suppress insulin secretion, and this, coupled with an increase in counterregulatory hormones, predisposes them to ketonemia.

While breastfeeding, intercurrent infection and excessive weight loss may exacerbate LKA [1, 2, 6, 8]. Our patient had a 14.2% (8.8 kg) weight loss within 3 weeks, which is more than the recommended safe target for weight loss (.5-1 kg per week), placing her at risk for malnutrition and electrolyte imbalances. Hence, the combination of an unmonitored ketogenic diet regimen, drastic weight loss, and possible mastitis in our nonobese patient may have contributed to the development of severe LKA.

Even after refeeding, in lactating women, the body continues to rely on fat and protein as energy sources, rather than immediately shifting to carbohydrate oxidation, indicating that dietary carbohydrates are probably prioritized to mammary glands to maintain the production of milk [10]. Factors such as the severity of the mother's condition and the availability of feeding alternatives for her baby should be considered when allowing the mother to continue breastfeeding. Most of the reported patients recovered within 24 hours, with no difference in recovery times regardless of whether they continued to breastfeed [8].

The aim of the treatment is to reverse ketoacidosis by establishing calorie replacement. Although no deaths have been reported, 9 of the 26 reported patients needed ICU care [1, 3-5, 7]. Nevertheless, regardless of the severity of acidosis, the successful reversal of ketoacidosis with glucose replacement was reported in all patients [1-8]. The majority of the reported patients received IV dextrose as calorie replacement in the treatment of LKA [8]. Aside from calorie replacement, correction of dehydration and electrolyte imbalances, and treatment of the precipitating factor such as infection, are equally important. Similar to most reported cases, our patient made a complete recovery within 24 hours despite presenting with severe acidosis and needing ICU care. The use of IV sodium bicarbonate in patients with severe acidosis and IV insulin to suppress ketogenesis was not associated with early resolution of the ketoacidosis and may potentially worsen electrolyte imbalances in patients with associated refeeding syndrome [1, 7, 8]. Finally, the maintenance of an adequate calorie intake and dietary counseling after recovery is vital to prevent recurrence of LKA [5].

In summary, our case illustrates a rare diagnosis of LKA that was rapidly reversed with early recognition and management. Self-prescribed weight-loss regimens such as ketogenic diets are commonplace among lactating women who may be pressured to lose their pregnancy weight, putting them at risk of LKA. Hence, it is important that nursing mothers are advised to ensure proper nutritional intake, including sufficient carbohydrates, while continuing to breastfeed their newborns.

## Learning Points

- Lactating women are at risk of lactation ketoacidosis because of the increased calorie demand during breastfeeding, particularly if there is concomitant starvation, such as that observed in ketogenic diet regimens.
- Lactation ketoacidosis can be severe; however, it is a rapidly reversible condition if diagnosed and managed promptly. The mainstay of treatment is adequate calorie replacement.
- All lactating women should receive nutritional education to prevent lactation ketoacidosis.

## Contributors

All authors made individual contributions to authorship. M.K.B., S.R.V., S.S.P., and T.S. were involved in the diagnosis and management of this patient. M.K.B. drafted and prepared the manuscript. S.R.V. and S.S.P. supervised and provided additional revisions of the manuscript. All authors reviewed and approved the final draft.

## Data Availability

Original data generated and analyzed during this study are included in this published article.

## Abbreviations

D10%: dextrose 10%
ICU: intensive care unit
LKA: lactation ketoacidosis
